# Rituximab resistant pemphigus vulgaris successfully treated with obinutuzumab

**DOI:** 10.1016/j.jdcr.2025.10.065

**Published:** 2025-11-08

**Authors:** Marjolein A.J. Hiel, Gilles F.H. Diercks, Maria C. Bolling, Jeroen Bremer, Joost M. Meijer, Barbara Horváth

**Affiliations:** a Department of Dermatology, University of Groningen, University Medical Center Groningen, Center of Expertise for Blistering Diseases, European Reference Network for Rare Skin Diseases (ERN SKIN), Groningen, the Netherlands; b Department of Pathology and Medical Biology, University of Groningen, University Medical Center Groningen, Groningen, the Netherlands

**Keywords:** anti-CD20 monoclonal antibody, Anti-rituximab antibodies, Obinutuzumab, Pemphigus Vulgaris, Rituximab resistance

## Introduction

Pemphigus vulgaris (PV) is an autoimmune bullous disease characterized by flaccid blisters and erosions of the skin and mucous membranes. Rituximab is the first-line treatment.^1^ While most patients exhibit excellent clinical responses to rituximab, some experience incomplete remission or relapse.^2^^,^^3^ The precise mechanism underlying this phenomenon can be diverse, but 1 mechanism is the development of anti-rituximab antibodies, leading to rituximab resistance.^2^ Obinutuzumab, a third-generation humanized anti-CD20 monoclonal antibody (mAb), has shown promising results in overcoming rituximab resistance in B-cell malignancies.^4^ However, its efficacy in rituximab-resistant PV has yet to be elucidated. We report a series of 3 patients with rituximab-resistant PV with good clinical response to obinutuzumab.

The diagnosis of PV was established based on its characteristic clinical and histopathological findings, intercellular IgG and/or C3 deposits on direct immunofluorescence, and the presence of serum anti-desmoglein 1 (DSG 1) and anti-desmoglein 3 (DSG 3) IgG detected by Enzyme-Linked Immunosorbent Assay.

## Report

**Fig 1:**
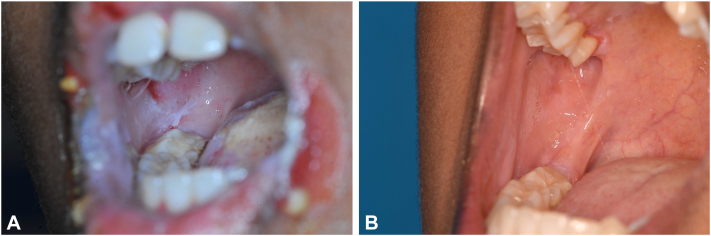
Clinical features of mucocutaneus pemphigus vulgaris before and after treatment with obinutuzumab in patient 1. **A,***White* plaques with blister roofs in the oral mucosa, along with rhagades at the oral commissures at presentation. **B,** Complete remission off therapy 47 months after presentation.

**Fig 2:**
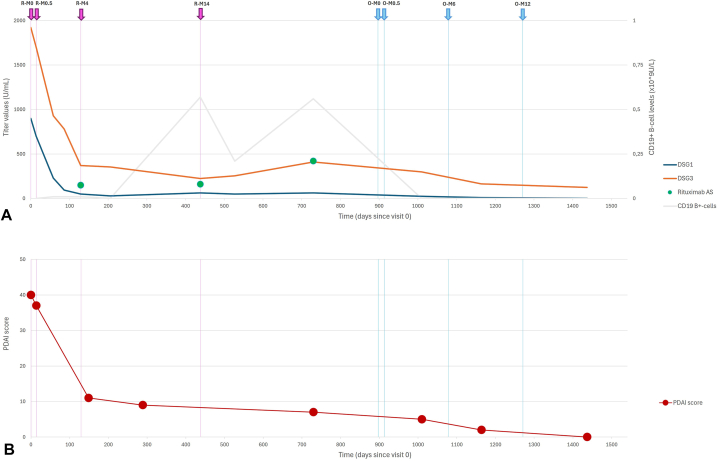
Immunological and clinical trends in Patient 1 during rituximab treatment and after switch to obinutuzumab. **A,** Immunological trends, including DSG 1 and DSG 3 antibody levels, anti-rituximab antibody levels, and CD19+ B-cell levels. **B,** Clinical course as measured by the PDAI score. *M*, Month; *O*, Obinutuzumab; *PDAI*, Pemphigus Disease Area Index; *R*, rituximab.

Patient 1, a 12-year-old girl with a 2-year history of mucocutaneous PV (mcPV) presented with erosions and flaccid blisters on the skin and oral mucosa (Fig 1, *A*), corresponding to a Pemphigus Disease Area Index (PDAI) score of 40 (Fig 2, *B*). She achieved partial remission after 2 rituximab administrations (1000 mg at Month [M]0 and 700 mg at M0.5) combined with oral (1 mg/kg/day) and topical corticosteroids, tapered within 20 weeks. Although antibody titers declined, they remained high (DSG 1: 230 U/mL, DSG 3: 930 U/mL). B cells, on the other hand, were completely depleted after M0 (0.0 × 10^9^/L), but increased to 0.01 × 10^9^/L following M0.5 (Fig 2, *A*). Consequently, the third rituximab dose (1000 mg) was advanced from M6 to M4, at which time anti-rituximab antibodies were detected at a level of 150 AU/mL. The patient’s clinical symptoms remained unchanged. She subsequently developed neutrophilic leukopenia, potentially as a side effect of rituximab, leading to postponement of the fourth rituximab dose from M12 to M14. The patient’s skin lesions had fully resolved; however, oral lesions persisted. Laboratory analysis at that time revealed increased anti-DSG 1 and 3 titers, along with B-cell repopulation (0.56 × 10^9^/L) and a high titer of anti-rituximab antibodies (420 AU/mL) (Fig 2, *A*). Due to the absence of B-cell depletion and rising anti-rituximab antibody levels, treatment with obinutuzumab was initiated with doses of 1000 mg administered at M0, M0.5, M6, and M12. After 3 months, there was significant clinical improvement, and after 18 months, the patient achieved complete remission with a PDAI score of 0 (Figs 1, *B* and 2, *B*). During a 6-month follow-up, the patient remained free of side effects, with no lesion recurrence.

Patient 2, a 57-year-old woman with a 2-year history of mcPV was treated with rituximab in combination with oral and topical corticosteroids. After 2 administrations (1000 mg at M0 and M0.5), she experienced an exacerbation with elevated anti-DSG 1 and 3 antibody levels, and incomplete B-cell depletion. After receiving rituximab 1000 mg (M6) and intravenous immunoglobulin (2 g/kg) for 5 days, the symptoms improved, and the patient achieved partial remission. Laboratory results showed a decrease in DSG 1 and 3 antibody levels, and complete B-cell depletion for the first time.

Due to difficulty achieving B-cell depletion, the patient received 1000 mg rituximab at M12, followed by an additional 1000 mg at M12.5. Nevertheless, she remained in partial remission and developed anti-rituximab antibodies (110 AU/mL). Treatment with obinutuzumab was initiated with 1000 mg administered at M0 and M0.5, followed by 500 mg at M6 and M12, resulting in complete remission after 7 months. Remission was sustained for 18 months without any side effects, until the end of the follow-up period.

Patient 3, a 39-year-old woman with a 4-year history of mcPV showed poor response to 2 cycles of rituximab (first: 2 × 1000 mg and 2 × 500 mg; second: 2 × 1000 mg) in combination with oral and topical corticosteroids. Laboratory analysis, post-rituximab, revealed incomplete B-cell depletion and high antibody titers against rituximab (>1000 AU/mL). Obinutuzumab was started with doses of 1000 mg administered at M0 and M0.5, followed by 500 mg at M6 and M12. The patient achieved complete remission at M0.5, which was sustained through the end of the follow-up at M24, with no side effects observed throughout.

The demographics and clinical characteristics of the patients are presented in Table I.

**Table I:** Demographics and clinical characteristics of identified patients

Case	Age (y), sex	Diagnosis	Prior rituximab dosage	Anti-rituximab antibodies (AU/mL)	Obinutuzumab dosage	Current disease status
1	12, F	mcPV	1000 mg (M0)	420	2 × 1000 mg (M0 and M0.5) 2 × 1000 mg (M6 and M12)	Complete remission
			700 mg (M0.5)			
			1000 mg (M4)			
			500 mg (M14)			
2	57, F	mcPV	2 × 1000 mg (M0 and M0.5)	110	2 × 1000 mg (M0 and M0.5) 2 × 500 mg (M6 and M12)	Complete remission
			2 × 1000 mg (M6 and M12)			
			1000 mg (M12.5)			
3	39, F	mcPV	Cycle 1:	>1000	2 × 1000 mg (M0 and M0.5) 2 × 500 mg (M6 and M12)	Complete remission
			2 × 1000 mg (M0 and M0.5)			
			2 × 500 mg (M6 and M12)			
			Cycle 2:			
			2 × 1000 mg (M0 and M0.5)			

*M*, Month; *mcPV*, mucocutaneous pemphigus vulgaris.

## Discussion

As a first generation chimeric anti-CD20 mAb, rituximab may trigger the formation of human anti-chimeric antibodies against its murine fragment, preventing the drug from binding to B-cells,^5^ leading to an accelerated B-cell reconstitution and rituximab resistance. Accordingly, testing for anti-rituximab antibodies is essential when the rituximab response is poor with no B-cell depletion. In such situations, obinutuzumab may provide a solution, as its fully humanized structure reduces—but does not eliminate—the risk of anti-drug antibody formation, and its higher binding affinity and distinct binding geometry enable more effective and sustained B-cell depletion compared with rituximab.^6^ Alternative approaches such as plasma exchange, intravenous immunoglobulin, and corticosteroid pulse therapy are less favorable, as they do not achieve B-cell depletion and therefore provide only transient benefit with poorer long-term outcomes.

Obinutuzumab may thus represent an effective alternative for patients with PV who are resistant to rituximab due to anti-rituximab antibodies, although its use and optimal dosing regimen in rituximab-resistant pemphigus vulgaris have not yet been established.

## Conflicts of interest

Hiel, Diercks, and Bremer have no conflicts to declare. Bolling reports fees from Krystal Biotech (advisory board, consultations), Chiesi (consultations) and Janssen-Cilag (payment for education development). All contracts were reviewed by the Board of Directors of the UMCG, and all fees were paid to the institution. JM reports fees from Argenx (Advisory Boards, Consultations), Sanofi (Consultation), Janssen-Cilag (Consultation). All contracts were reviewed by the Board of Directors of the UMCG, and all fees were paid to the institution. Horváth reports fees from Janssen-Cilag (Advisory Boards, Educational grants, Consultations, Investigator Initiative Studies), AbbVie (Advisory Boards, Educational grants, Consultations, Investigator Initiative Studies), Novartis Pharma (Advisory Boards, Consultations, Investigator Initiative Studies), UCB Pharma (Advisory Boards, Consultations), Leo Pharma (Consultations), Solenne B.V. (Investigator Initiative Studies), Celgene (Consultations, Investigator Initiative Studies), Akari therapeutics (Consultations, Investigator Initiative Studies), Philips (Consultation), Roche (Consultation), Regeneron (Consultation) and Sanofi (Consultation), Argenx (Advisory Boards, Consultations) and Boehringer-Ingelheim (Advisory Boards, Consultations). All contracts were reviewed by the Board of Directors of the UMCG, and all fees were paid to the institution.
